# Phagocyte Escape of *Leptospira*: The Role of TLRs and NLRs

**DOI:** 10.3389/fimmu.2020.571816

**Published:** 2020-10-06

**Authors:** Ignacio Santecchia, María Florencia Ferrer, Monica Larucci Vieira, Ricardo Martín Gómez, Catherine Werts

**Affiliations:** ^1^Institut Pasteur, Microbiology Department, Unité Biologie et Génétique de la Paroi Bactérienne, Paris, France; ^2^CNRS, UMR 2001 Microbiologie intégrative et Moléculaire, Paris, France; ^3^INSERM, Equipe Avenir, Paris, France; ^4^Université de Paris, Sorbonne Paris Cité, Paris, France; ^5^Laboratorio de Virus Animales, Instituto de Biotecnología y Biología Molecular, CONICET-Universidad Nacional de La Plata, La Plata, Argentina; ^6^Departamento de Microbiologia, Universidade Federal de Minas Gerais (UFMG), Belo Horizonte, Brazil

**Keywords:** leptospires, phagocytes, macrophages, neutrophils, platelets, TLR—toll-like receptor, NLR (NOD-like receptor), zoonosis

## Abstract

The spirochetal bacteria *Leptospira* spp. are causative agents of leptospirosis, a globally neglected and reemerging zoonotic disease. Infection with these pathogens may lead to an acute and potentially fatal disease but also to chronic asymptomatic renal colonization. Both forms of disease demonstrate the ability of leptospires to evade the immune response of their hosts. In this review, we aim first to recapitulate the knowledge and explore the controversial data about the opsonization, recognition, intracellular survival, and killing of leptospires by scavenger cells, including platelets, neutrophils, macrophages, and dendritic cells. Second, we will summarize the known specificities of the recognition or escape of leptospire components (the so-called microbial-associated molecular patterns; MAMPs) by the pattern recognition receptors (PRRs) of the Toll-like and NOD-like families. These PRRs are expressed by phagocytes, and their stimulation by MAMPs triggers pro-inflammatory cytokine and chemokine production and bactericidal responses, such as antimicrobial peptide secretion and reactive oxygen species production. Finally, we will highlight recent studies suggesting that boosting or restoring phagocytic functions by treatments using agonists of the Toll-like or NOD receptors represents a novel prophylactic strategy and describe other potential therapeutic or vaccine strategies to combat leptospirosis.

## Introduction

Leptospires are diderm bacteria belonging to the phylum Spirochetes and are classified as extracellular pathogens. These bacteria are responsible for a zoonosis with a worldwide distribution with a higher incidence in poor countries and tropical humid areas. Some animals, including rats and mice, are chronic carriers of leptospires in their kidneys, particularly in the lumen of the proximal tubules. They excrete the bacteria in the urine and contaminate the environment. Leptospires are found in water and soil and can infect all vertebrates, including mammals. Transmission occurs through transdermal or mucosal penetration of the bacteria, which first strongly adhere to skin and mucosal surfaces. Then, the bacteria reach the blood circulation and disseminate to all organs.

In terms of the symptoms and severity of the diseases caused by *Leptospira* spp., most *Leptospira* infections are asymptomatic. *Leptospira interrogans* are responsible for the most severe forms of leptospirosis in both humans and animals (1). In humans, the symptoms vary from a flu-like disease with fever, headaches, and muscular pains to more severe forms with icterus, hemorrhages, pulmonary or kidney insufficiency, requiring hospitalization. It was estimated in 2015 that in 5% of cases, leptospirosis led to multiorgan failure and accounted for 60,000 fatalities (2).

Compared to the saprophytic *L. biflexa* Patoc strain, which grows rapidly and is amenable to genetic manipulation, *L. interrogans* are difficult bacteria to study because of their extended generation time (approximately 18 h), the difficulty of obtaining mutant strains, and the fact that *in vitro* passaging quickly leads to the loss of virulence. In addition, more than 350 serovars have been described based on the immunogenicity of lipopolysaccharide (LPS), the major antigen of leptospires. Serovar diversity complicates diagnostics and constitutes one of the main barriers to obtaining a universal vaccine against leptospirosis (1).

One of the first lines of defense of the innate immune system is comprised of antibacterial components present in the serum. The complement system is a complex set of proteolytic cascades and opsonins that aim to directly destroy pathogens or target them for destruction by immune cells, such as macrophages (MΦ). This system is considered a nonspecific innate mechanism (3, 4). In addition, preimmunized hosts have a repertoire of antibodies that specifically target a pathogen for elimination and destruction. Therefore, both antibodies and other opsonins are of special importance for destroying pathogens through neutralization and engulfment by professional phagocytes, such as MΦ and neutrophils. The phagocytic function is mediated by several membrane-associated receptors on the cell surface, such as scavenger receptors (5, 6) and Fc receptors, which are exclusively dedicated to the recognition of the fragment crystallizable (Fc) regions of antibodies (6, 7). In addition, upon infection, phagocytes produce reactive oxygen species (ROS), such as nitric oxide (NO), and other potent antimicrobial compounds that participate in pathogen elimination.

Pattern recognition receptors (PRRs) recognize microbial-associated molecular patterns (MAMPs). They are essential, evolutionarily conserved structures shared among microbes but are not found in the host and include viral or bacterial nucleic acids and lipopolysaccharide. PRRs also recognize endogenous molecules associated with cellular damage (DAMPs) that are produced upon microbial infection, for example, (8). PRRs are expressed on both immune cells and nonimmune cells and include members of the membrane Toll-like receptor (TLR) and the cytosolic NOD-like receptor (NLR) families (9). MAMP recognition by a PRR triggers a signaling cascade leading to activation of transcription factors such as NF-κB and IRF3 involved in the production of cytokines, chemokines, and antimicrobial peptides, which leads to the activation and recruitment of phagocytes, such as neutrophils, MΦs, and dendritic cells (DCs), at the site of infection. The resulting inflammation not only may lead to pathogen destruction but also, if uncontrolled, may be deleterious for the host, such as the “cytokine storm” observed in septic patients. PRR activation also results in the expression of costimulatory molecules at the surface of MΦ and DCs that are important for antigen presentation to naive T cells and the onset of adaptive immunity.

Several studies have explored the role of phagocytes during leptospiral infection. In part I of this review, we will address the cellular biology of leptospire infection *in vitro* and *ex vivo* with a focus on the role of opsonization, the intracellular localization of leptospires, and cell death. We will also highlight *in vivo* studies suggesting the limited role of phagocytes in leptospires. In part II, we will recapitulate what has been published about leptospire recognition by or escape from TLR and NLR proteins. In part III, we will present recent studies suggesting that boosting TLR or NLR responses may help the host combat leptospirosis.

## Part I—Phagocytes; Poor Foes for *Leptospira*

### *In Vitro* and *Ex Vivo* Studies of the Role of Phagocytes and Scavenger Receptors

#### Macrophages (MΦ)

**Figure 1:**
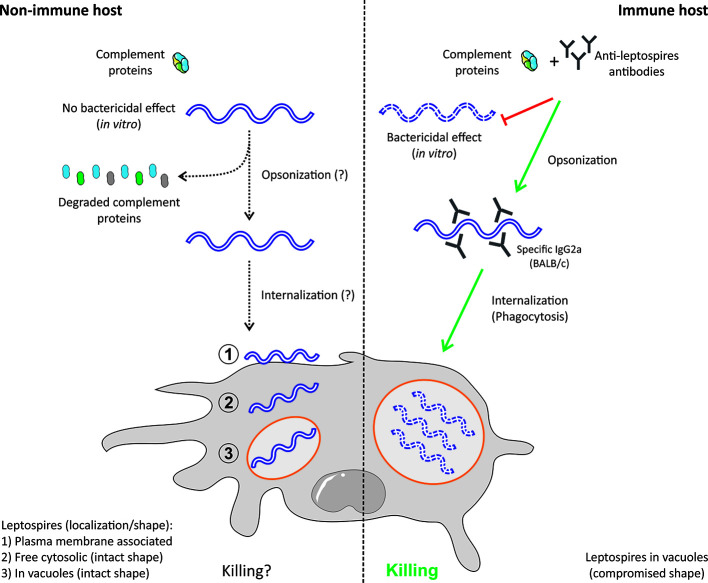
Effects of opsonization on leptospire survival in macrophages. *Leptospira interrogans* are resistant to nonimmune serum and have evolved diverse mechanisms to avoid the complement system. In contrast, immune serum exerts a bactericidal effect on leptospires. Preincubation of leptospires with immune serum containing anti-leptospires antibodies (Abs) leads to rapid internalization and killing of leptospires by macrophages. In this case, leptospires are exclusively found in vacuoles, in which they have compromised shapes. When leptospires are incubated with naive serum, their internalization seems to be slower, and several populations can be identified inside and outside macrophages: (1) plasma membrane-associated, (2) free in the cytosol, and (3) or in vacuoles. In this case, vacuolar leptospires do not seem to have a compromised shape (12, 17).

**Figure 2:**
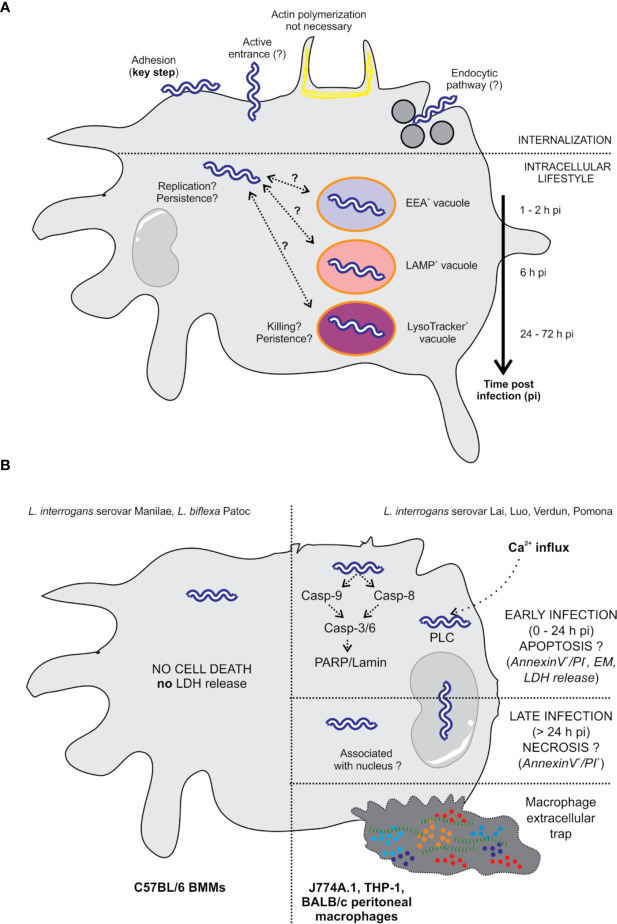
Intracellular localization of *L. interrogans* and effect on cell death. **(A)** The internalization of leptospires is highly dependent on adhesion to the cell surface (25). The entrance mechanism is not clearly established since it does not seem to involve actin polymerization, suggesting an entrance mechanism other than phagocytosis (18, 25, 26). Viability seems to be essential since formalin-fixed bacteria do not enter the cells (18). The endocytic pathway could potentially be involved in the internalization of leptospires since inhibition of this pathway in macrophages drastically reduced the number of intracellular bacteria (18). Once inside the cells, leptospires are found free in the cytosol in human and murine macrophages. In addition, some authors describe that leptospires are found in EEA1-, LAMP-, and LysoTracker-positive compartments using colocalization (20, 24). Colocalization seems to be extended over time, which may suggest the arrest of potential phagosomes/autophagosomes at several stages. In human cells, leptospires appear to be replicative (20). However, in murine cells, the overall number of leptospires seems to be constant or diminished over time (20, 24), with the unique observation that live bacteria are still found at 72 hpi, suggesting their persistence. **(B)** *L. interrogans* serovar Manilae does not induce macrophage cell death upon infection of BMMs obtained from a C57BL/6 background (24). *L. interrogans* serovars Lai, Luo, and Verdun induce cell death by apoptosis/necrosis upon infection of J774A.1, THP-1, and BALB/c peritoneal macrophages (18, 21–23). However, not all the studies have completely correlating results, and some controversies are revealed in the literature. Additionally, some studies report that leptospires are associated with the nucleus (18, 23).

This section will review the literature regarding the antibacterial effect of serum, the effect of complement antibody opsonization on the internalization of leptospires by MΦ (**Table 1** and **Figure 1**), the fate of leptospires in MΦ (**Table 2** and **Figure 2A**), and the complex data about the effect of *Leptospira* spp. on cell death (**Table 3** and **Figure 2B**).

**Table 1:** Opsonization of leptospires.

Leptospira spp	Host cells	Opsonization	Main findings (In vitro/Ex vivo or In vivo)	Ref
*L. interrogans* Icterohaemorrhagiae *L. biflexa* Doberdo	Guinea pig Mφ (*Casein/NaCl-elicited*)	NS	0.5 hpi: < 10% infected cells (high number of extracellular bacteria) 1–2 hpi: 30% infected cells; intracellular bacteria: conserved shape and no bactericidal activity. Cytosol-free and vacuole-associated bacteria (EM).	(10)
*L. interrogans* Icterohaemorrhagiae *L. biflexa* Doberdo	Guinea pig Mφ (*Casein/NaCl-elicited*)	Rat IgM and IgG, 40 days postimmunization. (naive guinea pig serum)	*L. biflexa* but not *L. interrogans* is affected by incubation with nonimmune serum. Mφ showed enhanced killing of IgG-preincubated leptospires that are associated with vacuoles, in which the bacterial shape is compromised (EM).	(11)
*L. interrogans* Copenhageni	BALB/c mice Mφ (*Thioglycolate-elicited*)	Rabbit anti-sera	No phagocytosis or killing was observed. Anti-sera opsonization led to phagocytosis and killing.	(12)
*L. interrogans* Icterohaemorrhagiae *L. biflexa* Patoc	Human blood monocytes and monocyte-derived Mφ	Nonimmune and immune serum	Immune serum is bactericidal towards leptospires *in vitro*. Leptospires opsonized with immune serum are internalized and killed (>90%) and show a compromised intracellular shape (EM).	(13)
*L. interrogans*	BALB/c and ddY mice Mφ (*Starch-elicited*)	Monoclonal IgG2a and IgG2b	*Ex vivo*: BALB/c and ddY Mφ phagocytize and kill leptospires. Preincubation with antisera increased internalization, killing, and association of leptospires with vacuoles. IgG2a but not IgG2b mediated uptake. *In vivo*: iv silica depletion led to increased mice susceptibility. Immunization with heat-inactivated leptospires or leptospiral LPS led to rapid blood clearance upon rechallenge.	(14)
*L. interrogans*	BALB/c mice Mφ (*Starch-elicited*)	NS	*Ex vivo*: pretreatment of Mφ with leptospiral or *E. coli* LPS enhanced phagocytic and bactericidal activity. Leptospiral LPS triggered ROS production. *In vivo*: iv-injected leptospiral LPS accumulated in spleen, liver, and lymph node Mφ.	(15)
*L. interrogans* Copenhageni	Zebrafish embryos	NS	2 hpi: leptospires trigger migration of Mφ. Upon infection, Mφ contain leptospires independent of opsonization. Infected macrophages presented a different morphology. 24 hpi: leptospires were located in hematopoiesis-associated tissue.	(16)

Mφ, macrophages; NS, nonspecified; EM, electron microscopy; pi, postinfection; ROS, reactive oxygen species; iv, intravenous.

**Table 2:** Intracellular localization and fate of leptospires.

Leptospira spp.	Host cells	Main findings	Technical remarks	Ref
*L. interrogans* Icterohaemorrhagiae	Vero and J774A.1 cell lines	*L. interrogans* showed rapid internalization (20 min) that was lost after a few *in vitro* passages. Delayed or impaired internalization (60* min*) of formalin-fixed and highly passaged strains. *L. biflexa* was extracellularly adherent. Slow internalization (60 min). Cytochalasin D: does not block internalization.	No gentamicin protection assay Double staining of extra- and intracellular bacteria	(18)
*L. interrogans* Lai *L. biflexa* Patoc	Vero and J774A.1 cell lines	Leptospires attached to host cells. Increased adherence in J774.1 compared to Vero cells. EM: “phagosome” and “lysosome” were observed. FCM: actin remodeling during infection. Colocalization of leptospires with the marker LAMP-1.	No gentamicin protection assay Important were controls missing	(19)
*L. interrogans* Lai (virulent), Pomona Luo (avirulent)	Human (THP-1 and primary Mφ) and murine (J77A.1, naive peritoneal cells and BMMs)	High adherence to all cell types. Murine cells: Leptospires were controlled (↓ CFU and viability). Membrane-associated bacteria showed a compromised shape. Increased colocalization with lysosomal markers over time. Human cells: Leptospires replicated (↑ CFU and viability). Cytosolic bacteria showed intact shapes. Replicative bacteria (CFU). Decreased colocalization with lysosomal markers over time.	No gentamicin protection assay Contradictory results compared to (21)/(22) Infection protocol ND	(20)
*L. interrogans* Lai, Luo *L. biflexa* Patoc	Vero and J774A.1 cell lines	*L. interrogans* [Lai (virulent) and Luo (avirulent)] but not *L. biflexa* adhered to cells (↑ adherence to Mφ). Lai and Luo observed inside of Vero and J774A.1 cells in the membranous compartment (EM).	No gentamicin protection assay	(23)
*L. interrogans* Lai	THP-1 and J774.1 cell lines	Leptospires triggered ROS production in both cell lines with no difference upon infection. Leptospires were intracellular (FCM) and associated with membranes (EM) in both cell lines.	Contradictory results compared to (20) Missing noninfected & nonstained controls	(21)
*L. interrogans* Manilae *L. biflexa* Patoc	C57BL/6 BMMs	Early - mid (1–6 hpi): intracellular leptospires colocalized with EEA-1 and LAMP-1. Saprophytic but not pathogenic leptospires showed a compromised shape. Infection with pathogenic strains led to delayed recruitment of cathepsin D and colocalization with LysoTracker. Late (24 hpi): only pathogenic strains were intracellularly membrane-associated (EM). Viable pathogenic bacteria were recovered in EMJH 24 and 48 hpi.	No gentamicin protection assay Double staining of extra- & intracellular bacteria	(24)
*L. interrogans* Manilae (low & high passage) *lmb216*/*ligB* mutants	C57BL/6 BMMs	The high-passage strain, *ligB*, and *lmb216* (absent in *L. biflexa*) showed reduced adhesion and infection. Expression of LigB and Lmb216 in *L. biflexa* increased adhesion and infection of Mφ. Cytochalasin D partially reduced but did not block internalization.		(25)
*L. interrogans* Lai	THP-1 and J774.1 cell lines	Leptospires observed in phagosomes in both cell lines (EM).	Contradictory results compared to (20)	(22)
*L. interrogans* Pomona	Bovine PBMCs	More cells were infected with the virulent strain than the passage-attenuated strain. Production of IL-1β, TNF-α, and IL-10. Infection and colocalization with lysosomal markers were not affected by cytochalasin D.	No gentamicin protection assay	(26)

EM, electron microscopy; EFM, epi-fluorescence microscopy; FCM, fluorescence confocal microscopy; ROS, reactive oxygen species; EMJH, Ellinghausen-McCullough-Johnson-Harris culture media; pi, postinfection; Mφ, macrophages; EMC, extracellular matrix components; ND, nondescribed.

Host cells: THP-1, human monocyte cell line; J774.1, murine macrophage-like cell line; BMMs, bone marrow-derived macrophages; Vero, monkey kidney epithelial cells; PBMCs, peripheral blood mononuclear cells. In red, technical issues and/or studies that should be interpreted with caution because of a lack of controls and/or internally contradictory results. In green, techniques of interest.

**Table 3:** *Leptospira-*induced apoptosis and cell death.

Leptospira spp	Host cells	Main findings	Technical remarks	Ref
*L. interrogans* Icterohaemorrhagiae	Vero and J774A.1 cell lines	Live pathogenic leptospires induced DNA fragmentation in Mφ. The saprophytic and avirulent strain did not induce DNA fragmentation.	No gentamicin protection assay Noninfected controls missing	(18)
*L. interrogans* Lai, Luo *L. biflexa* Patoc	Vero and J774A.1 cell lines	Subcellular “lesions” upon infection with Lai (virulent) and Luo (avirulent) (EM). Surprisingly, Lai was occasionally associated with nuclei. Live and UV-killed Lai and Luo induced apoptosis (annexin V+/PI-) in Vero cells and necroptosis (annexin V+/PI+) in Mφ. Both live and UV-killed serovars produced a similar phenotype.	No gentamicin protection assay Noninfected controls missing	(23)
*L. interrogans* Lai *L. biflexa* Patoc	BALB/c naive peritoneal Mφ J774A.1, A549, HUVEC, and ECV304 cell lines	Infection of Mφ and A549 cells induced cell death (LDH release-, MOI- and time-dependent) Lai (virulent) but not Patoc induced apoptosis (2–6 hpi) and later induced (> 12 hpi) necroptosis. Caspase-3, -6, -8, and -9 were activated upon infection with Lai but not with Patoc. Lai induced cleavage of PARP and Lamin A/C. FADD levels increased upon infection of Mφ. Induction of apoptosis was also observed in primary naive peritoneal Mφ.	No gentamicin protection assay Noninfected controls missing	(27)
*L. interrogans* Lai, Pomona Luo (avirulent)	Human (THP-1 and primary Mφ) and murine (J77A.1 and peritoneal BALB/c Mφ	Lai (virulent) induced increased apoptosis in murine Mφ compared to that in human Mφ (0–24 hpi). Lai induced necroptosis in murine Mφ (8–48 hpi).	No gentamicin protection assay	(20)
*L. interrogans* Manilae *L. biflexa* Patoc	C57BL/6 BMMs	No cell death was associated (no LDH release) with *L. interrogans* or *L. biflexa* infection.	Positive control for LDH release	(24)
*L. interrogans* Lai	THP-1 and J774.1 cell lines	Infection triggered accumulation of p53 and H2AX foci in a ROS-dependent manner. Leptospire infection arrested the cell cycle. Apoptosis/necrosis induced upon infection of Mφ.		(21)
*L. interrogans* Pomona	Bovine PBMCs	Infection triggered the formation of bMETs independently of the virulence of leptospires.		(26)

Mφ, macrophages; EM, electron microscopy; PI, propidium iodine; UV, ultraviolet; ND, nondescribed; pi, postinfection; ROS, reactive oxygen species; IP_3_, inositol-3-phosphate; bMETs, bovine macrophage extracellular traps; ⪼.

Host cells: THP-1, human monocyte cell line; J774.1, murine macrophage-like cell line; BMMs, bone marrow-derived macrophages; Vero, monkey kidney epithelial cells; PBMCs, peripheral blood mononuclear cells; A549, adenocarcinomic human alveolar basal epithelial cells; HUVECs, human umbilical endothelial cells; ECV340, human bladder epithelial cells; PMNs, polymorphonuclear cells. In red and green, technical remarks that mitigate or confirm the authors’ findings.

##### Role of Opsonization in Leptospire Survival and Interaction With MΦ

Early studies performed from 1960 to the mid-1980s showed that *in vitro* incubation with nonimmune serum does not exert bactericidal activity against pathogenic leptospires (11, 13, 28, 29). These studies described that pathogenic, but not saprophytic, leptospires were resistant to complement-induced destruction (11, 28, 29). More recently, other studies have confirmed this observation and shown that leptospires are resistant to complement (30–32). Nevertheless, *in vitro* incubation with immune serum exerts bactericidal activity on saprophytic and pathogenic leptospires (13, 28, 29). Given the importance of serum components and antibodies in MΦ function, several studies have addressed their role in phagocyte function. When leptospires were opsonized with immunoglobulin G (IgG), guinea pig peritoneal MΦ‘ bactericidal activity was enhanced, and leptospires were found in membrane compartments of MΦ with a compromised shape (11). In line with this study, Cinco et al. showed that elicited peritoneal MΦ from guinea pigs had no antibactericidal activity if the eliciting leptospires were not opsonized (10). Free nonopsonized leptospires were found in the cytosol or in membrane compartments. In contrast, opsonized leptospires were found in membrane compartments with compromised shapes. Furthermore, Wang et al. reported that human monocytes and MΦ only take up leptospires if they are opsonized with immune but not with normal serum (13). Opsonized leptospires were also found inside vesicles with altered shapes (13). Using peritoneal MΦ from BALB/c mice, Tu et al. reported that phagocytosis is only observed when leptospires are pretreated with rat anti-leptospiral serum (12). In contrast, Isogai et al. showed that murine MΦ do not need opsonization to take up leptospires, although opsonization drastically increases the uptake efficiency (14). Uptake seems to be mediated by IgG2a rather than IgG2b, and opsonization increases the rate of uptake of membrane-associated leptospires (14). The data presented in this section are recapitulated in **Figure 1** and **Table 1**.

Interestingly, in studies that did not use opsonizing conditions that resemble the physiology of a nonimmune host, it was found that motility and the endocytic pathway but not actin remodeling were involved in the internalization of pathogenic leptospires (18, 25, 26), suggesting their escape from phagocytic internalization. Interestingly, motility has been shown to alter phagocytosis of other pathogens, such as *Pseudomonas aeruginosa*, in both murine and human MΦ (33). Since motility constitutes one of the few virulence factors of leptospires [reviewed in (34)], this would represent a potent evasion mechanism that deserves further investigation.

##### Intracellular Localization and Fate of Leptospires

Phagocytosis requires the engagement of different receptors that trigger the engulfment of the pathogen (5). It is an actin-mediated process (35) that delivers the engulfed cargo into the lysosomal compartment for degradation. In addition, phagocytized particles can be used for antigen presentation. The phagocytic pathway is complex and involves many partners. Several players (36) such as Rab GTPases (37, 38) and phosphatidylinositol kinases (PIK) (39) are essential to the formation and maturation of the phagosome and for antigen presentation (40). Numerous proteins serve as hallmarks of key steps. For example, early phagosomes are characterized by the presence of early endosome antigen 1 (EEA1) and Rab5 (38, 41, 42). Rab5 then further recruits Rab7 in the so-called late phagosome, which is also characterized by the presence of lysosomal-associated membrane proteins (LAMPs). The latter are required for lysosomal fusion and acidification (43). Lysosomal acidification to a pH of 4.5–5 is mediated by a V-type H^+^ ATPase (44), allowing for the optimal functioning of lysosomal enzymes such as cathepsins and proteases. Many pathogens have evolved strategies to block or inhibit phagocytic killing (45, 46). For example, Rab proteins are selectively targeted to the benefit of the pathogen by two intracellular pathogens, *Mycobacterium tuberculosis* (47) and *Salmonella enterica* (48). Whether leptospires subvert MΦ killing functions is still unknown.

However, many studies have studied the fate of leptospires when they are incubated with MΦ or epithelial cells. In both cell types, virulent leptospires are rapidly internalized. In contrast, avirulent (high culture passage) and saprophytic strains were adherent to the cell surface and less likely to be internalized. The endocytic pathway and viability but not actin polymerization were shown to be involved in the internalization of leptospires (18). In contrast, other studies found that virulent (serovar Lai) and avirulent (serovar Luo) *L. interrogans* but not *L. biflexa* rapidly adhere to MΦ and epithelial cell surfaces (23). Both serovars were observed inside MΦ by electron microscopy (EM) in membrane-associated compartments (23). Furthermore, both serovars were found to adhere to and be internalized by murine and human MΦ (cell lines and primary cells) (20). In human cells, leptospires appear in a replicative form, are free in the cytosol, and conserve their helical shape (20). However, leptospires partially colocalize with the lysosomal marker LAMP-1 after infection (20). On the other hand, in murine cells, leptospires are killed, and they are found in membrane compartments with compromised round shapes (20). In murine cells, leptospires are delivered into LAMP-positive compartments over time with a plateau at 48 h postinfection (hpi) (20). Although leptospires seem to be killed by murine MΦ, MΦ still contain live and viable leptospires 72 hpi (20). Surprisingly, the same research group showed 3 years later using the same settings and strains that upon infection of human cells, leptospires are located in phagosomes (21), which suggests that the careful interpretation of both articles is necessary (**Table 2**).

In a set of different studies, Toma et al. used bone marrow-derived MΦ from a C57BL/6 background and infected them with virulent (low passage) and avirulent (high passage) *L. interrogans* serovar Manilae or with saprophytic *L. biflexa* serovar Patoc (24, 25). Both pathogenic and saprophytic leptospires were found to be intracellular (24). Infection was shown to be highly dependent on virulence and adhesion through at least 2 proteins, LigB and Lmb216, which is a putative adhesin present only in pathogenic species (25). Notably, this study confirmed that actin polymerization does not play a role in the internalization process (25).

Interestingly, *L. biflexa* are killed and show a compromised shape, whereas pathogenic bacteria conserve their shape and are not killed (24, 25). Both EEA-1 and LAMP were found to colocalize with leptospires after infection (24). However, the phagosomal protease cathepsin D showed delayed recruitment kinetics toward the pathogenic strain (24). Furthermore, only the saprophytic strain was found to colocalize almost completely with the lysosomal marker LysoTracker (24). In contrast, pathogenic leptospires showed a decreasing level of colocalization with lysosomal markers (24). This set of observations seems to indicate the arrest of the maturation of the lysosome after internalization of pathogenic leptospires. To support these observations, confocal microscopy images of intracellular leptospires were obtained, and EMJH cultures were performed to assess viability. Not surprisingly, saprophytic but not pathogenic leptospires were killed at 24 hpi (24). Moreover, live pathogenic leptospires were observed for at least 48 hpi (24). More recently, using MΦ of bovine origin, Nagel et al. observed that leptospires are intracellular and that their internalization is dependent on virulence and is independent of actin polymerization (26). Intracellular leptospires were found in LAMP- and LysoTracker-positive compartments (26). This set of observations suggests that leptospires are not internalized *via* actin-dependent phagocytosis and that the arrest of the maturation of lysosomes could take place upon infection with pathogenic leptospires.

Interestingly, opsonized leptospires are found in vacuoles in MΦ from resistant (murine) or sensitive (human and guinea pig) hosts, revealing the central role of MΦ in peritoneally immunized hosts regardless of their sensitivity to the disease (10–14). Once they are inside MΦ, leptospires colocalize with phagosomal/lysosomal pathway markers (13, 26, 36). This could indicate that leptospires are located in the phagosomal compartment; however, the persistent colocalization of these markers may also indicate the arrest of the phagocytic pathways or the presence of persistent forms. Such is the case for the intracellular pathogen *Listeria monocytogenes*, which switches to a vascular lifestyle once it is inside host cells (49). In this state, *L. monocytogenes* persists in the lysosomal environment, which favors both survival and asymptomatic carriage of this pathogen (49). This can have an impact on intracellular survival, as shown by Li et al. (20) and Toma et al. (24), which showed that the overall number of intracellular leptospires seems to be either decreased or constant. In any case, it seems that there are a few persistent bacteria inside cells that are not cleared even 72 hpi.

In conclusion, there are several questions about aspects of the literature about MΦ and leptospires that are still difficult to interpret or should be considered with caution due to the lack of controls, experimental caveats, or internally contradictory results (**Tables 2**, **3**). Once they are inside of murine MΦ, leptospires are localized in membrane-associated compartments (20, 24, 26). However, in human cells, the localization of leptospires is not clear since conflicting results indicate that leptospires are either free in the cytosol (20) or found in phagosomes (21) (**Table 2**). This difference could be due to the culture conditions of leptospires or the state of the infected MΦ, allowing leptospires to be internalized inside vacuoles or to remain in the cytosol. Another possibility to explain the discrepant results could be that experiments performed at different time points could reflect that leptospires are either trapped in a vacuole or have had time to escape from a vacuole. Whether leptospires modulate intracellular vacuole trafficking to benefit their survival during an infection, similar to what is observed for other pathogens such as *Staphylococcus aureus* (50), remains to be studied.

##### Apoptosis and Cell Death

Programmed cell death (PCD) is a crucial physiological and homeostatic process that can be triggered under different stress conditions (51). It occurs through different mechanisms, including apoptosis, necroptosis, and pyroptosis. Each of these mechanisms has its own signaling pathway and physiological role (51). Apoptosis is an immunologically silent form of cell death in which cells undergo a noninflammatory type of programmed cell death (PCD). Apoptotic cells are engulfed by phagocytes, and any threat is hence eliminated. On the other hand, although they are mechanistically different, both necroptosis and pyroptosis are inflammatory types of PCD. *In fine*, PCD can be considered a host defense mechanism against pathogens that may use the host as a reservoir. Therefore, it is not surprising that during infection, pathogens modulate cell death pathways to their advantage (52, 53). This section aims to present studies that describe MΦ cell death during infection with pathogenic leptospires. The main findings are summarized in **Table 3** and **Figure 2**.

Some studies reported that leptospires were associated with the nucleus in some cells, suggesting a particular killing mechanism (18, 23). Li et al. showed that both live and dead leptospires trigger apoptosis/necrosis, which is surprising to some extent (23). Furthermore, *L. interrogans* serovar Lai triggers apoptosis early upon infection, although necrosis is the most prevalent form of cell death in MΦ at late stages postinfection (20). In addition, infection seems to trigger the nuclear accumulation of p53 and DNA damage (21). A mechanism was proposed in murine MΦ by which *L. interrogans* serovar Lai but not *L. biflexa* serovar Patoc activates caspase-3- and caspase-8-mediated PCD (27). This type of PCD seems to be mediated by a leptospiral phospholipase involved in the accumulation of intracellular Ca^2+^ that is associated with apoptotic cell death (22). Unfortunately, some of these results are conflicting (see **Tables 1**, **4**), making their interpretation complex. On the other hand, infection with *L. interrogans* serovar Manilae does not induce LDH release (24), suggesting that this strain does not induce cell death.

**Table 4:** Neutrophils.

Leptospira spp	Host cells	Main findings	Ref
*L. interrogans* Copenhageni, Canicola, Icterohaemorrhagiae Hebdomadis and Parameles *L. biflexa* Patoc*, L. illini*	SUS and RES Rat PMN (starch-/peptone-elicited, Ficoll-purified)	Blood of infected rats was positive for ROS production (chemoluminescence) after leptospiral infection. SUS rats (low PMN function) were more sensitive to infection than RES rats (high PMN function). Live but not formalin-inactivated leptospires induced production of ROS. Virulent (V) strains triggered reduced ROS production compared to nonvirulent (AV) and saprophytic strains. Opsonization with complement & immune serum led to enhancement of ROS and complete bacterial killing	(54)
*L. interrogans* Copenhageni *L. biflexa* Patoc	Human neutrophils (Ficoll)	Infection triggered an increase in CD11b expression, adhesion to collagen, formation of mixed platelet–leucocyte aggregates, activation of NF-κB (production of IL-8 (TLR2-dependent) and IL-6) and NLRP3-derived IL-1β, neutrophil chemotaxis, and increased AXL expression. Saprophytic but not pathogenic leptospires triggered production of ROS and were phagocytized. Infection had an anti-apoptotic effect and did not activate MPO	(55)
*L. interrogans* Icterohaemorrhagiae *L. biflexa* Patoc	Human neutrophils (Ficoll)	*L. interrogans*: even in the presence of 10% serum, no killing or ingestion occurred. Adherent and associated with neutrophils but also extracellular. *L. biflexa*: even in absence of serum, it was killed and phagocytized, Intracellular and localized in vacuoles.	(56)
*L. borgpetersenii* Hardjo *L. interrogans* Copenhageni and Pomona, *L. biflexa* Patoc	Bovine neutrophils	Pathogenic and saprophytic leptospires triggered the formation of NETs and production of ROS, RNS, IL-1β, IL-8, MIP-1α, and TNF. Stronger responses were triggered by live versus heat-killed leptospires. No bactericidal effect of PMN on pathogenic and saprophytic leptospires. Naive and immune serum did not alter the formation of NETs.	(57)
*L. interrogans* Copenhageni *L. biflexa* serovar Patoc	Human neutrophils	Leptospiral infection induced the release of HBP, increased intracellular Ca^2+^ and ROS production, and a nonapoptotic effect. High HBP serum levels were found in leptospirosis patients. The lipoproteins Lsa63 and LipL45 were responsible for HBP release and increased endothelial permeability *in vitro*. Lsa63 led to increased vascular permeability *in vivo*.	(58)
*L. interrogans* Copenhageni	Human neutrophils	LIC11207 was conserved in pathogenic strains and prevented neutrophil apoptosis *in vitro*. LIC11207 was expressed in hamster kidneys colonized by leptospires & was recognized in serum from leptospirosis patients.	(59)
*L. interrogans* Copenhageni	Human neutrophils	Incubation of leptospires with neutrophils inhibited MPO activity but increased elastase activity. LipL21 and LipL45 but not Lsa63 or LPS are responsible for the inhibition of MPO activity.	(60)
*L. interrogans* Hardjoprajitno *L. biflexa* Patoc	Human neutrophils	Pathogenic and saprophytic leptospires were sensitive to ROS. MPO (neutrophil primary granule component) did not have bactericidal activity toward leptospires.	(61)
*L. interrogans* Icterohaemorrhagiae	Human and murine neutrophils	Human and mouse neutrophils did not phagocytize leptospires and produced low amounts of ROS and RNS. Macrophages and not neutrophils were the main infiltrating cells in a mouse model.	(62)
*L. interrogans* Copenhageni and Manilae *L. biflexa* Patoc	Human neutrophils	MOI and the viability-independent formation of NETs were increased in pathogenic versus saprophytic leptospires. Bactericidal activity of neutrophils depended partially on NETs. Pathogenic leptospires degraded NET-associated DNA. In mice, neutrophils played a role in the control of leptospires through NETs in the acute and chronic phases.	(63)
*L. interrogans* Copenhageni	Human neutrophils	Stimulation of neutrophils with leptospiral LPS is PAF-dependent and produced ROS. Rabbit but not human platelets aggregated in response to leptospiral LPS.	(64)

PMN, polymorphonuclear; ROS, reactive oxygen species; CD11b, cluster of differentiation 11b; IL, interleukin; MPO, myeloperoxidase; NETs, neutrophil extracellular traps; RNS, reactive nitrogen species; MIP, macrophage inflammatory protein; TNF, tumor necrosis factor; IFN, interferon; TFG, tumor growth factor; HBP, heparin-binding protein; LPS, lipopolysaccharide; PAF, platelet-activating factor.

More recently, infection with bovine MΦ was shown to trigger the formation of macrophage-extracellular traps (METs) (26), which have also been observed during neutrophil infection (63). This effect seems to be independent of the virulence status of leptospires since a low or high number of passages lead to similar formation of METs (26). Although this phenomenon is considered a particular form of PCD, some people have reported that after the release in NETS, neutrophils remain alive (65).

In fact, there seems to be a clear difference between the Lai and Icterohaemorrhagiae serovars compared with the Manilae strain in terms of the induction of PCD upon MΦ infection. However, the referenced studies did not use the same infection model (66, 67). This could be the basis of the major difference between strains, as in the case of *M. tuberculosis*, in which virulent strains only escape apoptosis mediated by infected MΦ (68). The use of MΦ of different origins (cell lines, primary bone marrow-derived, peritoneal) and from different hosts (guinea pig, mouse, rat and human) as well as the different species/serovar/strains of leptospires makes it very difficult to formulate a global interpretation. Furthermore, currently, it is widely accepted that even though they constitute a valuable resource for research, cell lines are also a major source of variability and reproducibility issues (69). In addition, most studies did not perform gentamicin assays (**Table 2**), which is a caveat when studying the internalization of bacteria and leads to the questioning of the interpretation of some data, which are to be considered with caution (**Table 2**). Therefore, additional work is required to better understand whether different leptospire strains may behave differently and/or whether species-specific aspects of the intracellular environment may explain some of the controversial data (**Table 2** and **Figure 2**).

#### Neutrophils Recognize Leptospires but Are Poor Phagocytes

Neutrophils are key cells that act against extracellular pathogens through three major mechanisms: (i) phagocytosis, which usually requires opsonization beforehand; (ii) degranulation, which involves the release of an arsenal of cytotoxic molecules stored in granules; and (iii) the release of extracellular traps (ETs, or NETs, in this case). Although neutrophils are short-lived, their life span is significantly extended under infectious and inflammatory conditions (70). Moreover, neutrophils can produce ROS through NADPH activation and the release of pro-inflammatory cytokines involved in the activation, regulation, and effector functions of innate and adaptive immune cells (71).

**Figure 3:**
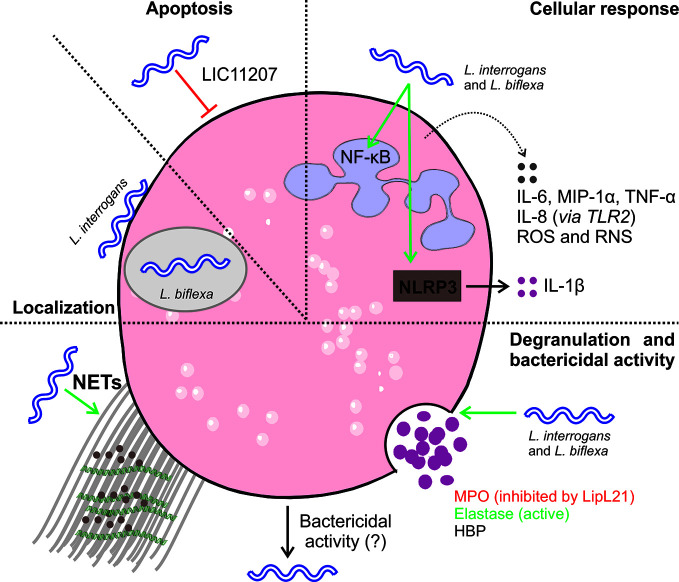
Interaction of *L. interrogans* and neutrophils. Upon infection, leptospires are observed at the neutrophil surface or intracellularly in vacuoles. Live pathogenic leptospires seem to be resistant to internalization unless they are opsonized. In contrast, saprophytic leptospires are rapidly internalized and killed (13, 54, 55). Leptospires do not trigger apoptosis (58, 59), and they have an anti-apoptotic effect on infected cells (55). Intracellular uptake of leptospires triggers cellular responses characterized by the production of antibacterial components such as reactive oxygen species (ROS) and reactive nitrogen species (RNS) (54, 55, 57, 58, 61, 62, 64). In addition, infection triggers TLR2-mediated production of IL-8 and NLRP3-dependent production of IL-1β (55). Moreover, infection triggers the production of other antibacterial mediators, such as cytokines and chemokines (55, 57), but does not induce or inhibit neutrophil myeloperoxidase (MPO) activity (55, 60). Cellular responses tend to be more robust for live leptospires than heat- or formalin-inactivated leptospires (54, 55, 60, 63). Moreover, infection triggers the production of neutrophil extracellular traps (NETSs) (57). The bactericidal activity of neutrophils has not always been observed, and it seems to be dependent on the sources of cells, the serovar used, and the use of opsonizing antibodies (13, 55, 57, 60).

Early *in vitro* studies of *Leptospira* phagocytosis by neutrophils showed some partially contradictory results (**Table 4** and **Figure 3** neutrophils). However, phagocytosis by human polymorphonuclear leucocytes (PMN) of both virulent and avirulent *Leptospira* spp. required the presence of specific immune serum and complement components (72), while other studies showed that only saprophytic leptospires were phagocytized in the absence of serum, and pathogenic leptospires were not phagocytized by human PMN even in the presence of serum (13). Interestingly, it was also reported that avirulent leptospires showed increased phagocytosis by human PMN compared to virulent leptospires, and phagocytosis was markedly enhanced after opsonization (54).

In a recent EM analysis (55), most pathogenic leptospires were found on the neutrophil surface and were not phagocytized. In contrast, saprophytic leptospires were taken up. Intracellular ROS levels correlated with leptospire uptake. Altogether, it seems that pathogenic leptospires can avoid or significantly reduce their uptake by human neutrophils, but the precise mechanisms and *in vivo* relevance involved are unknown.

Regarding degranulation, it has been shown that both virulent and avirulent *Leptospira* spp. are killed by both primary and secondary granule contents. The primary (azurophilic) granules, which contain myeloperoxidase (MPO), heparin-binding protein (HBP), defensins, and other antimicrobial peptides (AMPs), showed the highest microbicidal activity (61). Remarkably, it has been recently shown that the *L. interrogans* outer membrane protein LipL21 is a potent inhibitor of neutrophil MPO (60). This heme-containing peroxidase enzyme, which is mainly expressed in neutrophils, catalyzes the formation of ROS intermediates in the presence of hydrogen peroxide and halides, including hypochlorous acid (HOCl), a major effector of microbial killing by neutrophils. Interestingly, MPO deficiency results in exaggerated inflammatory responses and affects neutrophil functions, including cytokine production (73). In addition, the leptospiral proteins Lsa63 and LipL45 can induce release of the HBP protein into the extracellular space (58), which in turn can induce endothelial cell cytoskeletal rearrangements, leading to disruption of cell barriers and increased vascular permeability (58, 74). The role of MPO and HBP in the pathogenesis of leptospirosis would be further clarified by using animal models deficient in each neutrophil component.

Concerning the formation of NETs, it was demonstrated that *Leptospira* spp. were able to induce NET release in human neutrophils and that the bacterial number, pathogenicity, and viability were relevant factors for NET release induction, whereas bacterial motility was not (63). Interestingly, although NETs reduced leptospire viability, pathogenic but not saprophytic *Leptospira* spp. exerted nuclease activity and degraded DNA, suggesting that pathogenic leptospires may counteract this microbicidal mechanism (63). The formation of NETs was also observed when *Leptospira* spp. were incubated with bovine neutrophils, although the amounts were lower than those induced by *E. coli* (57), suggesting that the *in vivo* role of NETs in *Leptospira* dissemination may be less important than that of other bacteria. More recently, it was reported that both avirulent and virulent *Leptospira* spp. triggered neutrophil responses involved in migration, including the upregulation of CD11b expression and adhesion to collagen. In addition, both species activated the NF-κB and inflammasome pathways (see part II-4) and increased the levels and release of the pro-inflammatory chemokine IL-8 and the cytokines IL-6 and IL-1β. As expected with PMN activation, leptospires delayed neutrophil apoptosis (55).

#### Platelets

Platelets play a well-recognized role in hemostasis and thrombosis, and it is now broadly accepted that platelets also have an important role in inflammation and immune responses (75). Platelet immunological functions include the secretion of functional mediators such as cytokines and chemokines (76) as well as direct interactions with immune cells (77). In addition, platelets may function as direct scavengers in bacterial infections (78, 79). To accomplish such diverse functions, in addition to all the components that are critical to guarantee hemostasis, platelets express several PRRs (80), namely, TLRs, complement, and Fc-γ receptors (81). Human platelets express 10 members of the TLR family, which are functional PRRs that not only sense microbes but also trigger platelet effector responses that modulate the innate immune response (82). In addition to TLRs, platelet cell adhesion molecules, such as glycoprotein Ib (GPIb), P-selectin, and CD40L, allow intimate contact with inflammatory neutrophils and monocytes through binding to their counterreceptors αMβ2, PSGL-1, and CD40 (83). Moreover, platelets also interact (touch-and-go interactions) with leukocytes, including DCs, T cells, and B cells (84, 85). These associations favor crosstalk between platelets and neutrophils, resulting in bidirectional activation of both cell types and amplification of the inflammatory response (65).

Early studies reported that LPS from pathogenic *Leptospira* spp. leads to aggregation, degranulation, and lysis of rabbit platelets *in vitro* (86). In contrast, it was reported that the pathogenic leptospiral immunoglobulin-like protein B (LigB) binds to the C-terminus of the fibrinogen αC domain, inhibiting fibrin clot formation and human platelet adhesion and aggregation (87), while leptospiral proteins containing the von Willebrand factor type A domain induce hemorrhage in leptospirosis by competitive inhibition of vWF/GPIb-mediated platelet aggregation (88, 89). Curiously, in human platelets, aggregation seems to occur only when platelets are in a mixture with PMN previously primed with leptospiral LPS but not when they are in platelet suspensions alone (64). In this regard, it has been shown by flow cytometry that *Leptospira* spp. induce the formation of neutrophil-platelet mixed aggregates (55), a fact that partially explains the thrombocytopenia observed in patients. It is clear that more studies about the effects of *Leptospira* on platelets are needed, including bacterial uptake, activation and viability (90).

#### Dendritic Cells

DCs are professional antigen-presenting cells, and their activation links the innate immune recognition of pathogens to the triggering of adaptive immunity. DCs express many different PRRs, including the carbohydrate receptor DC-SIGN of the C-type lectin family, which recognizes high-mannose glycans. DC-SIGN binds intercellular adhesion molecule-3 (ICAM-3) and ICAM-2 on T cells, promoting the adhesion of DCs to naive T cells.

One study showed that live virulent or attenuated strains of *L. interrogans* serovar Pyrogenes and Autumnalis induced the maturation of monocyte-derived DCs obtained from healthy blood donors *in vitro*. Leptospiral infection also triggered TNF and IL-12 cytokine production, both of which are important for T cell activation (91). The authors demonstrated that leptospires bind *in vitro* and *in vivo* to DC-SIGN through surface carbohydrates (91). However, DC-SIGN requires other PRRs to trigger cytokine production. Which PRRs are involved in cytokine production induced by *Leptospira* infection and whether leptospires are killed by DCs and properly processed for antigen presentation have yet to be investigated in both human and murine cells.

### *In Vivo* Role of Macrophages, Neutrophils, and Platelets During Leptospirosis

#### *In Vivo* Studies of Macrophages

One approach to studying the role of MΦ *in vivo* involves the depletion of these cells and the observation of the outcome of infection. Silica depletion *via* the intravenous (iv) route enhanced the susceptibility of mice to leptospiral infection independently of preimmunization with leptospires (14). Furthermore, intravenous (iv) immunization of undepleted mice with heat-killed leptospires or leptospiral LPS was found to have a positive effect on blood clearance, highlighting the role of phagocytes (14). More recently, using the zebrafish embryo model, Davis et al. showed that iv infection triggers active and rapid migration of MΦ to the site of infection, where they internalize leptospires (16). Interestingly, at this stage of development, there are no antibodies present in the embryo, which suggests that no opsonization or complement opsonization is required for internalization of the leptospires in these embryos (16). More recently, using clodronate depletion of MΦ, our groups showed that MΦ partially controls leptospires in mice, with effects observed at both the acute phase of uncontrolled initial infection (17) and at the chronic phase (92). In addition, in a mouse model, it was recently observed that MΦ but not neutrophils appeared to be the major infiltrating anti-*Leptospira* phagocytes in the kidneys during leptospirosis (62). Therefore, although they play a limited role in nonimmunized hosts, MΦ are important cellular components of the immune response required to control leptospires.

#### *In Vivo* Studies of Neutrophils

It is accepted that human leptospirosis causes neutrophilia, which correlates with severity (93–96), and the pathogenic serovar is involved (97). Moreover, some neutrophil molecules, such as neutrophil gelatinase-associated lipocalin (NGAL) and HBP, have been proposed as early markers of acute leptospirosis (58, 98). In leptospirosis patients, enhanced expression levels of TLR2, CD15 (94, 99), and TLR4 (99) were found on neutrophils. In contrast, low expression levels of the AMP LL37 cathelicidin (active on leptospires) were found in leptospirosis patients (95). Curiously, CD62L and CD11b levels were found to be similar to those of controls (94, 99). The discrepancies between the abovementioned *in vitro* studies (55) may be explained by several factors, including the number of circulating bacteria. In a mouse model, it was observed that although neutrophil depletion significantly increases the early leptospiral loads in blood (63), depletion did not modify the overall course of the disease (62, 63, 100). Unfortunately, mice and humans have important differences regarding neutrophils (as well as platelets) (101). Therefore, the real importance of neutrophils and the mechanisms of leptospiral escape remain to be determined in patients with leptospirosis.

#### *In Vivo* Studies of Platelets During Leptospirosis

Platelet activation and dysfunction have been reported in leptospirosis patients (102, 103). In addition, thrombocytopenia is a frequent clinical finding, particularly in severe leptospirosis cases, and is associated with a worse prognosis and occurrence of hemorrhages (80, 104–106). There is no consensus about the underlying mechanisms of the decrease in platelet numbers during leptospirosis. The hypotheses include (i) disseminated intravascular coagulation (DIC), (ii) toxin-mediated platelet death, (iii) impaired production of platelets, (iv) platelet overactivation and consumption, and (v) autoimmune platelet clearance. While some studies reported negative results for DIC in leptospirosis patients (107) as well as in guinea pig studies (108, 109), other studies found evidence of DIC in some leptospirosis patients (103). Thrombocytopenia in human leptospirosis is apparently not immune-mediated (110), and some studies suggest that it might result from activation (102, 110). Therefore, a comprehensive investigation is needed to determine the mechanisms of thrombocytopenia in the disease.

## Part II—Leptospires: Stealthy Pathogens That Escape Several PRR-Mediated Innate Responses

**Figure 4:**
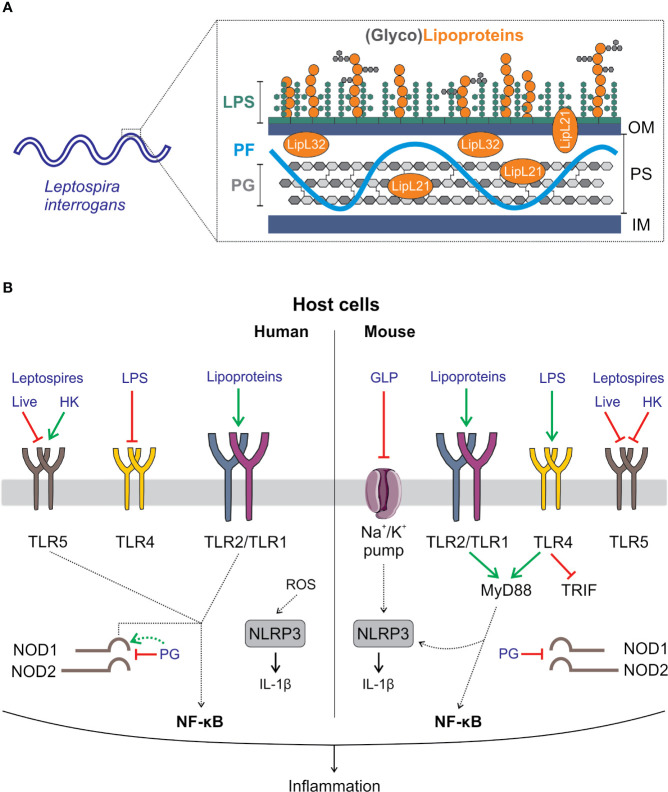
Interaction of *Leptospira interrogans* with the innate immune system. **(A)** *L. interrogans* are diderm bacteria. Their cell wall is composed of an inner membrane (IM) and an outer membrane (OM) containing lipopolysaccharide (LPS). In between the membranes, the periplasmic space (PS) contains the peptidoglycan (PG) layer, the periplasmic flagella (PF) that extends from each pole of the cell inside the PS and lipoproteins such as LipL32. Notably, leptospires contain in their OM a broad repertoire of lipoproteins and a glycolipoprotein (GLP) (111). **(B)** Host cells have a variety of mechanisms to detect microbes. One of them involves a subset of receptors collectively known as pattern recognition receptors (PRRs) that sense conserved and ubiquitously expressed microbial components. The latter are known as microbial-associated molecular patterns (MAMPs). Recognition of MAMPs by PRRs triggers the immune response. In blue, leptospiral MAMPs and MAMP sensing in human (left side panel) and mouse (right side panel) cells are shown. TLR2 is the main receptor for sensing leptospires, which is common in humans and mice. TLR2 heterodimerizes with TLR1 and detects triacylated lipoproteins. A major difference between sensitive humans and resistant mice is the specific sensing of the leptospiral LPS (113), a major leptospiral MAMP and virulence factor (34). Mouse TLR4 but not human TLR4 is able to detect it (114–116). However, the stimulation of mouse TLR4 by leptospiral LPS is only partial, since only the MyD88 pathway but not the TRIF pathway is activated. This results in only minimal production of antimicrobial components such as nitric oxide (117). Due to the presence of PF in leptospires, live leptospires also escape TLR5 recognition. However, once leptospires are killed by antimicrobial peptides that expose PF, leptospiral flagellins are recognized by human (and bovine) TLR5 but not by mouse TLR5 (118). Leptospires have been described to induce activation of the NLRP3 inflammasome in both humans and mice. In mice, activation of the inflammasome requires 2-step signaling involving LPS and lipoprotein signaling through TLR4 and TLR2, respectively, in combination with the downregulation of a Na/K pump (a danger signal) by GLP (119). In humans, NLRP3 activation is mediated by bacteria-induced ROS (120). Finally, PG from leptospires escapes host recognition by NOD1 and NOD2 due to the lipoprotein LipL21, which is tightly attached to the PG. In addition, human but not murine NOD1 can detect leptospiral PG in the absence of LipL21 (121).

The high motility of leptospires is conferred by two atypical “endoflagella” that are present at each pole of the bacterium and extend within the periplasm. Their spiral shape is due to the peptidoglycan mesh, forming a thin layer close to the inner membrane. The membranes are rich in lipoproteins (111). In contrast with that of other spirochetes, the leptospiral outer membrane is covered in lipopolysaccharide (LPS) (112). LPS, lipoproteins, and peptidoglycan are conserved components of bacteria but are absent from the host and are recognized by the innate system through PRRs from the TLR and NLR families (9). Here, we aim to recapitulate the more striking features of leptospiral MAMP recognition by PRRs expressed by phagocytes from different hosts (**Figure 4**). For a more extensive review of this topic, see (112).

### TLR2 Recognition of Lipoproteins

The most striking feature of innate recognition of leptospires is their potent lipoprotein-induced TLR2 activation (112, 114, 122–124). It was first shown that LipL32, the major lipoprotein of leptospires, is a TLR2 agonist in human macrophages and hamster ovary cells (114) and in mouse renal tubular proximal cells (125). We also determined that the recognition of leptospires occurred through dimers of TLR2 and TLR1 (115) specific for triacylated lipoproteins that are present in leptospires (111). More recently, it was shown that leptospires also stimulate pig and bovine fibroblasts through TLR2 (122, 123). The abundant lipoproteins present in the membranes of leptospires easily explain their potent TLR2 activity, at least *in vitro*. However, the regulation of their expression and posttranslational modifications may lead to immune evasion *in vivo* (111). Hence, despite being responsible for cellular activation and inflammation *in vitro*, TLR2 triggering is not essential to mouse defense. Indeed, in contrast with TLR4 knockout (KO) mice, TLR2KO mice do not die from experimental leptospirosis (116, 126). However, activation of TLR2 in addition to TLR4 contributes to host protection in mice by triggering cytokine secretion and leptospire-specific IgG, NO, and IFNγ secretion (116). In addition, we recently showed that the inflammatory response of PMNs infected with *L. interrogans* was dependent on TLR2 in human neutrophils (55).

Notably, we have also highlighted the potential protective role of TLR2 in the human defense against *Leptospira*. Indeed, single nucleotide polymorphisms (SNPs) in TLR2 and TLR1, which are known to modulate susceptibility to infectious diseases, have been linked to increased susceptibility to developing severe leptospirosis in Argentina (124). However, the Argentinian cohort of patients was small, and the SNP found in TLR2 has not been associated with increased susceptibility to leptospirosis in a population from the Azores Islands (127).

### TLR4 Recognition of LPS

LPS is a macromolecule composed of three sections: (i) a carbohydrate part responsible for the antigenic properties of LPS, (ii) a central core and KDO region, and (iii) lipid A, consisting of a disaccharide moiety linked to lipid chains that anchor LPS in the outer membrane. Lipid A, also known as “endotoxin,” is recognized by the TLR4/MD2 complex and is responsible for the toxic properties of LPS.

We first showed that the LPS of leptospires is not recognized through TLR4, as expected in human MΦ (114). However, it is recognized by murine TLR4 (115). The structure of leptospiral lipid A has been deciphered by the Raetz group and showed several modifications, including a methylated phosphate group and four amide-linked acyl chains, compared to the structure of classic lipid A from *Enterobacteria*, and these modifications result in the lack of reactive phosphate groups on each side of the disaccharide (113). These modifications potentially explain the lack of human TLR4 recognition, which is more stringent than its murine counterpart. Using deficient mice, it was shown that TLR4 is a major PRR involved in the control of leptospires (116, 128). The species specificity of TLR4 recognition most likely explains the differences in the severity of leptospirosis, which can be fatal in humans but asymptomatic in mice that are considered chronic carriers of *Leptospira* (34).

In addition, we recently showed that in mouse macrophages, TLR4 recognition of leptospiral LPS is not complete (117). Only one arm of the signaling pathway is triggered. Indeed, TLR4 has two adaptors: MyD88 at the cell surface and TRIF in the endosome. MyD88 signaling results in cytokine and costimulatory molecule expression, whereas TRIF leads to production of type I interferons and nitric oxide (NO) (117). Leptospiral LPS only stimulates the MyD88 pathway but does not stimulate the TRIF pathway, and as a consequence, whole bacteria induce only minimal antimicrobial NO and IFN-ß production. This mechanism may participate in bacterial evasion and potentially explains why LPS is a virulence factor (129) and why pathogenic leptospires succeed in reaching the mouse kidney despite TLR4 recognition. We showed that the escape mechanism is linked to the presence of a complete O antigen in LPS and tightly bound lipoproteins, which together impair the interaction of LPS with CD14, an important coreceptor required for TLR4 internalization and TRIF signaling (117). Indeed, purification protocols for leptospiral LPS (130) always lead to copurification of TLR2 agonists, suggesting the close structural association of LPS and lipoproteins (114). Moreover, we showed that the protein portion of lipoproteins but not the lipidic portion that signals through TLR2 is important for TRIF escape (117). In line with these data, a recent study showed that one strain of *L. interrogans* serovar Autumnalis responsible for the self-resolution of infection in a murine model possesses an LPS devoid of TLR2 activity (131).

### TLR5 Recognition of Flagellin

TLR5 is the receptor of bacterial flagellins, composing the filamentous part of flagella. We previously showed that live leptospires triggered production of equivalent amounts of IL1ß and IL6 cytokines in wild-type and TLR5KO bone marrow–derived MΦ, suggesting that murine TLR5 does not recognize leptospires (132). However, it has also been shown in human whole blood infected or stimulated with inactivated leptospires that a neutralizing TLR5 antibody decreased the cytokine response, suggesting that human TLR5 may recognize leptospires (133). Both observations were confirmed and explained by our recent study showing that live leptospires escape TLR5 recognition in both human and mouse macrophages, but leptospires that are either degraded by heating or killed by antimicrobial peptides such as the cathelicidins LL37 or bMap28 are sensed by human and bovine TLR5, although they are not detected or only barely detected by mouse TLR5. These results confirmed the hypothesis that the periplasmic localization of the endoflagella participates in immune evasion. From a therapeutic perspective, since *Enterobacteria* flagellin is a potent adjuvant (134), one may also hypothesize that escape from TLR5 could potentially limit the adaptive immune response to *Leptospira*. Moreover, we also highlighted that the structure of leptospiral FlaB flagellins, which are agonists of TLR5, is devoid of the variable portion that confers the antigenicity of flagellins, suggesting that leptospiral flagellins would not be efficiently recognized by antibodies; this adds a second layer of immune evasion.

### NOD1 and NOD2 Escape

NOD1 and NOD2 are cytosolic PRRs that recognize bacterial fragments of peptidoglycan called muropeptides, which are constantly released by the bacteria upon remodeling and synthesis of the cell wall (9). NOD1 and NOD2 are important for controlling invasive and extracellular bacteria (9). Activation of NOD receptors is also important for immune functions and the onset of adaptive immune responses (112). Indeed, in mice, NOD1 activation by the microbiota has been shown to be crucial for PMN killing of bacteria and fungi (135). In addition, we showed that NOD1 activation plays an important role in murine renal defense using a model of retro-urethral infection with uropathogenic *Escherichia coli* (136).

We showed that leptospires escape NOD1 and NOD2 recognition. We demonstrated that the lipoprotein LipL21 is tightly bound to peptidoglycan and impairs the release of muropeptides, therefore blocking NOD1/2 sensing (121). Moreover, we found a species-specific mechanism of NOD1 escape. Human and mouse NOD1 do not recognize exactly the same agonists (9). We showed that the peptidoglycan of *L. interrogans* is devoid of the murine NOD1 agonist but possesses fair amounts of the human NOD1 agonist, suggesting that bacteria in which LipL21 would be degraded or missing could also escape NOD1 sensing in mice but would still signal in human cells. This hypothesis has been confirmed using humanized NOD1 mice infected with the *L. interrogans* Manilae L495 and *lipL21* mutants (121).

The other role of LipL21, its potent inhibition of MPO activity in PMNs (60), is probably not linked to PG binding and NOD1 escape, since experiments with recombinant LipL21 led to similar observations as experiments with membrane fractions of whole bacteria (60).

Therefore, we hypothesize that LipL21 blocks muropeptide release (121) and, along with LipL45, inhibits MPO (60), most likely contributing to neutrophil escape and immune evasion. Because the leptospiral genome encodes more than 140 lipoproteins, most of them with unknown functions (111), we may speculate that other lipoproteins participate in immune evasion.

### NLRP3 Inflammasome Activation

The inflammasome is a platform for cytosolic proteins composed of NOD-like protein (NLRP), one or several adaptors such as the protein ASC, and pro-caspase 1. Inflammasome activation results in the secretion of pro-inflammatory IL1β. This cytokine is central to inflammation and is tightly regulated. In mice, two signals are required; the first signal leads to NF-κB activation and mRNA expression of pro-IL1ß and the components of the inflammasome, and the second signal activates the inflammasome through oligomerization of the NLRP proteins and recruitment of the ASC adaptor and pro-caspase 1, which itself is cleaved and activated to convert pro-IL1ß into mature IL1ß **** (9).

We have shown in murine bone marrow-derived MΦ that leptospires trigger the NLRP3 inflammasome through LPS and lipoprotein activation of TLR2 and TLR4 and downregulation of the potassium pump by glycolipoprotein (GLP) (119). We also showed that leptospiral activation of the inflammasome was mostly limited to NLRP3 and excluded other potential inflammasomes, such as NLRC4/NAIP5, which recognize flagellin. NLRP3-dependent IL1β secretion was confirmed in a recent study showing that doxycyclin reduced IL1β secretion in J774 murine MΦ cells infected with leptospires (137). Subsequently, it was shown that leptospires also trigger the NLRP3 inflammasome in the THP1 human MΦ cell line (120). The mechanism of activation was not investigated but revealed the contribution of ROS and cathepsin B, which was not found in mice.

### Species Specificity of PRR/Leptospire Recognition

One key point to consider is the species specificity of the innate immune responses to leptospiral MAMPs. Several studies presented in this review highlighted differences in the recognition of leptospires between different hosts that could contribute to species-specific sensitivity to the disease (**Figure 4**). For example, the intracellular fate of leptospires in phagocytes (20) as well as TLR4, TLR5 and NOD1 recognition (115, 117, 118, 121) differ between humans and mice. Recent studies on neutrophil and platelet activation by leptospires also suggest differences between humans and rats or rabbits (54–56, 64). In addition, we also highlighted a difference in TLR5 sensing in mice, in which TLR5 does not sense leptospiral flagellins, and cows, in which TLR5 can sense them, as in humans (118). Recognition of PRRs of leptospires by TLRs involved in nucleic sensing, including TLR3, TLR7, TLR8, TLR9, and those recognizing microbial RNAs and DNA, as well as other cytosolic sensors should also be considered.

## Part III—TLR/NLR Agonists Boost Phagocytic Responses Against Leptospires

**Figure 5:**
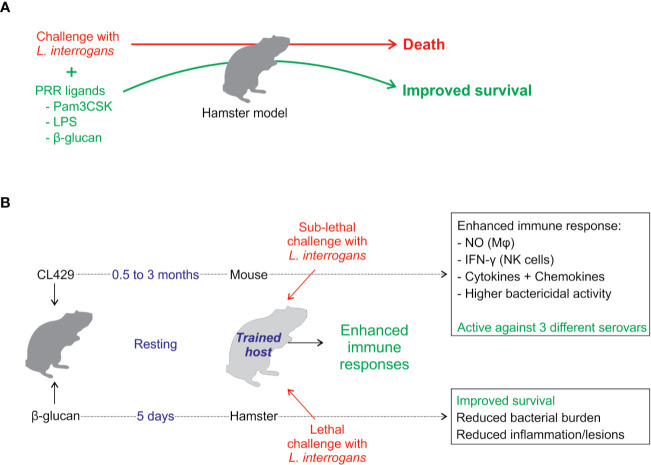
Enhancement of the immune system to better control leptospires. **(A)** Coinjection at the time of leptospiral infection of PRR agonists such as Pam3CSK (a TLR2 agonist) (138), β-glucan (a Dectin-1 agonist that potentially synergizes with TLR2) (139), or crude *Escherichia coli* LPS (a TLR2/TLR4 agonist) (126) leads to enhancement of hamster immune responses along with improved survival or delayed lethality and reductions in the number of survivors in tissue lesions, bacterial loads, and inflammation. **(B)** Recently, innate immune memory or trained immunity has been explored as a therapeutic strategy against leptospires. The TLR2/NOD2 agonist CL429 was used to treat mice before infection (17). After CL429 treatment, the animals were challenged with *L. interrogans*, and the treated group showed improved resolution of the acute phase of infection. Interestingly, CL429 treatment leads to enhanced antibacterial activity in macrophages characterized by enhanced production of antimicrobial compounds such as nitric oxide (NO). Moreover, the treatment has systemic effects that enhance the cellular response in the bone marrow and in spleen NK cells. Overall, this effect lasts for at least 3 months posttreatment, and *ex vivo* data suggest that it could be used to alleviate human leptospirosis. Another study used β-glucan (139), a main component of the fungal cell wall, to treat hamsters 5 days before infection. β-glucan pretreatment leads to reduced mortality and decreased bacterial burden in blood and target organs as well as a reduction in tissue lesions and inflammation (139).

Considering the previous section and the different PRRs involved in leptospire escape, the use of TLR and NLR agonists to boost or restore deficient leptospire responses is attractive. A few studies have shown that their use is effective in combating leptospirosis (**Figure 5**).

### Coinjection of TLR or PRR Agonists

Several recent studies from the Cao group used PRR agonists at the time of infection or 1 day prior to the infection, which showed some protective effects in hamsters, a sensitive animal model of leptospirosis (34).

First, it was shown that early expression of TLR2 (but not TLR4) was observed upon leptospiral infection in resistant BALB/c mice, whereas delayed expression was observed in sensitive hamsters (138). This confirmed a previous study from Matsui et al., suggesting that the early triggering of inflammatory mediators in mice infected with leptospires was protective, whereas the delayed response in hamsters could participate in a cytokine storm (140). Interestingly, anti-inflammatory IL-10 cytokine secretion was shown to be dependent on TLR2 in mice (140). The coinjection of hamsters with *L. interrogans* serovar Autumnalis and Pam3cysSK4, a synthetic agonist of TLR2, alleviated acute leptospirosis and improved the survival of hamsters, which showed reduced leptospiral loads and histopathological lesions in organs 3 weeks pi (138).

More recently, the same group investigated the effect of crude *E. coli* LPS administered in hamsters after infection with leptospires and showed an improved outcome in hamsters, which was correlated with increased inflammation levels (126). However, the use of nonrepurified crude *E. coli* LPS, which is known to activate both TLR2 and TLR4 (126, 141), made the interpretation of the relative contributions of TLR4 and TLR2 difficult. Nonetheless, an interesting observation is that upon leptospiral infection, cotreatment with *E. coli* LPS dramatically enhanced both NO secretion and MPO activity. However, *in vivo* pharmacological inhibition of NO did not impact the outcome due to LPS (119), suggesting that the enhanced or restored MPO activity (or activity of other mediators) could be responsible for protection. Interestingly, coinjections starting 1 day prior to the time of leptospiral infection with ß-glucan, a fungal cell wall component agonist of the Dectin-1 receptor known to synergize with TLR2, also increased early inflammation and improved survival (139).

### Prophylactic Use of TLR and NLR Agonists

By attempting to use *Lactobacillus plantarum*, a generally recognized as safe (GRAS) organism, as a platform to express leptospiral proteins to immunize sensitive C3H/HeJ mice through the oral route, we observed unanticipated protective effects. Indeed, after a 6-week regimen of intermittent oral gavage, it was shown that treatment with the parental *L. plantarum* strain protected mice from acute leptospirosis in the sensitive C3H/HeJ TLR4-deficient model (142). The symptoms of leptospirosis were alleviated, and 15 days postleptospiral challenge, the effect of *L. plantarum* was associated with the recruitment of macrophage-like cells in lymphoid organs and kidneys (142). *L. plantarum* did not prevent renal colonization, but the treatment reduced inflammation and renal fibrosis. Because these features were reminiscent of the newly introduced concept of “innate immune memory”, also known as “trained immunity,” during which a first infection or MAMP stimulation triggers metabolic and epigenetic reprogramming of MΦ and NK cells to cause them to more robustly respond upon a second challenge, we hypothesized that *L. plantarum* triggered such a mechanism. We tested the effect of two intraperitoneal injections of CL429, a dual TLR2-NOD2 agonist that was shown by others to recapitulate the protective and anti-inflammatory effects of oral *L. plantarum* in mice in a model of viral pulmonary infection (143). We demonstrated that CL429 pretreatment indeed induced an innate memory effect independent of B and T cells in MΦ from the peritoneal cavity but also in distant sites, such as the bone marrow and NK cells from spleen (17). CL429 pretreatment enhances the MΦ secretion of cytokines and NO and IFNγ production by NK cells. Notably, the CL429 effect lasted for at least 3 months posttreatment and was effective against the 3 *L. interrogans* serovars tested (17) (**Figure 5**). In addition, CL429 also triggered a trained immunity effect in human MΦ derived from monocytes of healthy blood donors (17). A recent study showed that ß-glucan, the prototypic MAMP used to induce trained immunity, was consistently effective in preventing severe leptospirosis in hamsters when administered 5 days prior to infection with *L. interrogans* serovar Lai strain 56601 (139) (**Figure 5**). ß-glucan protected 37.5% of hamsters from death and alleviated lesions in the liver, lungs, and kidneys (139).

## Conclusions: Current Gaps and Future Directions

Phagocytosis plays an important role in linking innate and adaptive immune responses (144). PRR activation is intricately connected to different phagocytic and platelet functions (65, 145, 146). The consequences of leptospiral PRR escape or recognition may directly influence phagocytic functions, which in turn may influence the shaping of adaptive responses.

MΦ and neutrophils barely control pathogenic leptospires, which largely escape phagocytosis. Strikingly, these responses vary between phagocytes and hosts. Indeed, although neutrophils are the primary professional bactericidal cells, they are unexpectedly even less active than MΦ in controlling *Leptospira* in naive hosts (62, 63, 94, 99). Bacterial adhesion and motility are key steps in the infection process, and pathogenic leptospires adhere to neutrophils (55, 56) and MΦ but only enter MΦ (18, 20–25). In contrast, it has been established that opsonization with immune serum significantly enhances bacterial uptake and killing in human and mouse MΦ (11–15, 147) but not in human PMN (55, 56), although this could be different for rat PMN (54). The effective response upon opsonization is an interesting point highlighting the fact that leptospires may avoid but do not deactivate phagocyte functions. Nevertheless, considering the newly acquired knowledge of trained immunity and the effect of priming phagocytes, the fact that many opsonization and internalization experiments have been performed with preactivation of cells with casein/NaCl, starch or thioglycolate (148) (**Tables 1**, **4**), which are all known to elicit increases in cells in the peritoneal cavity and to boost MΦ function, brings into question the physiological relevance of the corresponding data. Hence, a gap exists in the knowledge of the *in vitro* features of PRR escape in leptospires and the physiological consequences of phagocyte function for leptospires. The precise mechanisms and the consequences for adaptive responses remain to be studied. In addition, considering the host specificity of PRR recognition in leptospires, additional infection experiments using different animal models and primary cells are mandatory to assess the functions of phagocytes during leptospirosis.

Although many studies have been performed on MΦ/neutrophils, almost nothing is known about DC recognition of leptospires. Since the skin and mucosa are the first organs to be infected by leptospires, Langerhans DCs are the first antigen presenting cells to be in contact with leptospires. DCs should be crucial in alerting the host to the presence of invading leptospires through PRR sensing and in digesting the bacteria and presenting antigens to naive T cells. Since the adaptive immune response to leptospires is believed to mostly rely on a T cell–independent strong humoral response against LPS that is not durable (112), we may speculate that leptospires may somehow overcome the DC response. The process of DC recognition and antigen presentation of leptospires in different hosts is currently under investigation. Hopefully, this will also increase our understanding of the poor immune responses towards leptospires that allow for chronic renal carriage and shedding of leptospires. Whether leptospires trigger memory B and T cell immunity in relation with PRR recognition or escape is an important question that should be addressed in different hosts and, if possible, with primary cells.

Hopefully, an improved understanding of the immunobiology and function of phagocytes in the context of leptospirosis may help to exploit the ability of phagocytes to fight leptospires and aid in the development of novel therapeutics. Recent results presented here suggest that the use of PRR agonists targeting phagocytes could constitute a novel strategy to fight leptospirosis. Trained immunity using some PRR agonists seems to be a promising prophylactic tool to alleviate acute leptospirosis, but it remains to be tested in animals other than mice and hamsters, such as cattle. In addition, it also remains to be understood whether such strategies may be beneficial to shape a more robust adaptive cellular immunological response against *Leptospira*. In addition to prophylaxis, it will also be important to study whether such strategies could be used to treat active infections either at the acute phase during leptospiral dissemination in blood or at the chronic phase of renal colonization. These PRR-based strategies may also promote the design of novel and efficient vaccines to fight leptospirosis, a neglected re-emerging zoonotic disease for which only a few serovar-specific and short-lived vaccines are currently available.

## Data Availability Statement

All datasets presented in this study are included in the article/supplementary material.

## Author Contributions

IS, FF, MV, and RG contributed to the writing of part I. IS conceived the tables and did the figures. CW wrote the original draft. All authors contributed to the article and approved the submitted version.

## Funding

This work was supported by the Institut Pasteur grant PTR2017-66 (CW). This is part of the Ph.D. thesis of IS, a fellow from the PPU program with funding from Institut Carnot Pasteur Microbes et Santé. IS also received a 4^th^ year Ph.D salary from the Fondation pour la Recherche Médicale (FDT201805005258). This work was supported by grants PICT 2016-1740 (RG) and PICT 2016-2608 (MF) from the Agencia Nacional de Promoción Científica y Tecnológica (ANPCyT) and PPID X037 (MF) from UNLP, Argentina. The funders had no role in study design, data collection and interpretation, or the decision to submit the work for publication.

## Conflict of Interest

The authors declare that the research was conducted in the absence of any commercial or financial relationships that could be construed as a potential conflict of interest.

The handling editor declared a past co-authorship with several of the authors RG, MF.
